# The Mediator Effect of Meaningfulness on the Relationship Between Schizotypy Traits and Suicidality

**DOI:** 10.3389/fpsyg.2020.00493

**Published:** 2020-03-24

**Authors:** Shu-bin Li, Ding Liu, Xiao-yuan Zhang, Jiu-bo Zhao

**Affiliations:** Department of Psychology, School of Public Health, Southern Medical University (Guangdong Provincial Key Laboratory of Tropical Disease Research), Guangzhou, China

**Keywords:** meaningfulness, suicide, schizotypy, mediator effect, structural equation model

## Abstract

**Introduction:** Previous studies have found that schizotypy can predict suicide, and that meaningfulness has influential effects on suicidality in the general population. However, it is still not clear whether meaningfulness is associated with suicidality in individuals with schizotypy. The aim of this study was to assess the mediating effects of meaningfulness in the relationship between schizotypy and suicidality.

**Method:** We recruited 2,615 university students (including undergraduates and postgraduates; mean age = 18.35, *SD* = 0.81; males, 39%) to participate in our study, and used the Meaningful Life Measure (Chinese, revised) to assess their experiences of meaningfulness, the Suicidal Behaviors Questionnaire (Chinese version) to evaluate participants' suicidal thoughts and attempts, and the Schizotypal Personality Questionnaire to examine their personal schizotypal traits.

**Results:** Meaningfulness was found to be inversely related to the other two factors, and schizotypy was positively associated with suicidality. The correlation between schizotypy and suicidality was reduced when meaningfulness was included, which may suggest that meaningfulness can partly mediate this relationship, accounting for 55.47% of the association.

**Conclusions:** Prior research has suggested that assessing meaningfulness could provide more detailed information about suicide risk in individuals with schizotypy. Our study found that improving perceptions of meaningfulness can be an effective intervention in reducing suicide risk among people with schizotypy.

## Introduction

Suicide is a complex and important global public health problem (Turecki and Brent, 2016). Each year, nearly 800,000 suicide deaths occur worldwide, which makes it one of the leading causes of death (World Health Organization, 2018). Mental disorder is an important factor for suicide (Harris and Barraclough, 1997; Arsenault-Lapierre et al., 2004; Nock et al., 2010; Hoertel et al., 2015; Honings et al., 2016) and more than 90% of individuals who have committed suicide had had a diagnosis of mental illness at the time of death (Bertolote and Fleischmann, 2002), such as, for example, schizophrenia (Joiner et al., 2001; Hawton et al., 2005; Hor and Taylor, 2010). Schimanski et al. (2016) found that schizophrenia states can be non-specific indicators to suicide-related ideations and behaviors, and that schizotypy can still predict passive ideation even when the depression has been controlled.

Similarly, schizotypy is associated with an increased likelihood of suicide attempts. Schizotypy represents a phenotypic manifestation of putative vulnerability for schizophrenia-spectrum disorders, which means schizotypal individuals are at high risk of developing into full-blown psychosis (Nelson et al., 2013; Fonseca-Pedrero, 2017; Fonseca-Pedrero et al., 2017; Grant et al., 2018). The heterogeneity between schizotypy and schizophrenia lies in etiology, development and response of treatment (Kwapil et al., 2018; Lenzenweger, 2018). Conceptualized as a personality organization or trait, “schizotypy” can encompass similar neurocognitive, behavioral, and psychopathological problems as schizophrenia, and has the potential to develop into schizophrenia (Meehl, 1962; Kwapil et al., 2013). Therefore, schizotypy is prevailingly thought to be comprised of three basic factors, which are similar with the positive, negative, and disorganized dimensions of schizophrenia (Nelson et al., 2013). Positive symptoms are associated aberrant perceptual experiences, like magical ideation and referential thinking. Negative schizotypy is related to anergia, anhedonia, social disinterest, and flat affect (Kwapil et al., 2013, 2018). In a long-term follow-up cohort, 40% of the people with schizophrenia-spectrum disorders were found to have suicidal ideation, 23% had experienced suicidal attempts, and 6.4% had committed suicide (Fenton et al., 1997). The symptoms of schizotypal personality disorder even have been shown to predict the presence of lifetime suicide attempts after controlling for some risk factors, such as childhood abuse, and comorbid psychiatric diagnoses (Lentz et al., 2010). Moreover, Joiner et al. (2001) reported that people with schizotypal symptoms were more associated with self-hatred and suicidality, and that the combination of self-hatred and a diagnostic status of schizophrenia-spectrum symptoms may be a predictor of suicidality. The early intervention of suicidality targeting specific personality traits may be beneficial to decreasing suicide rates (Canal-Rivero et al., 2018).

There are many variables that can affect the relationship between schizotypy and suicidality, such as self-hatred (Joiner et al., 2001), depressive symptoms (Jahn et al., 2016), self-esteem (Jahn et al., 2016), and childhood abuse (Lentz et al., 2010). These negative experiences may be associated with the specific cognitive and interpersonal defects of individuals with schizotypy and they also tend to undermine these individuals' sense of value in respect of themselves and their lives. Frankl (1978) defined this emotional involvement of self-fulfillment and self-value as “meaning in life.” Meaningfulness is strongly related to depressive symptoms, but they have entirely different connotations: depressive symptoms focus on the assessment of negative emotions and external behavior performance whereas meaningfulness emphasizes the power of the inner self and the internal experience. In other words, the loss of meaningfulness comes about from many elements—not only depressive symptoms, but also traumatic events and inherent personality. Additionally, many studies have documented that life meaning has a beneficial effect on reducing suicidality (Edwards and Holden, 2001; Orbach et al., 2003; Kleiman and Beaver, 2013; Wilchek-Aviad, 2015). Focusing on the special personality disorder in schizotypy, therefore, meaningfulness can provide us more information on suicide precaution and intervention.

However, the current literature has yet to investigate whether life meaning is associated with suicidality in individuals with schizotypy. The purpose of the present study, therefore, is to explore whether the relationship between schizotypy and suicidality can be explained by meaningfulness. We hypothesize that meaningfulness can be a mediator of suicidality and schizotypy. Accordingly, we expect to find that meaningfulness will be inversely related to schizotypy and suicidality. In addition, we explore a model of the relationship among suicidality, meaningfulness, and schizotypy.

## Materials and Methods

### Participants

The participants in the study were 2,615 students (39% males, 99.9% freshmen) in the university, including both undergraduates and postgraduates. The sample had an age range of 17–25 years old (mean age = 18.35 years old, *SD* = 0.81 years). 99.9% were freshmen in university. Ten people have been diagnosed as depression previously but they are all haven't received psychotherapy for over 3 years. The other participants (99.6%) were free from psychiatric diseases, organic lesions, and drug/alcohol misuses, based on their self-reports. In addition, among all the participants, 92.8% were Han nationality; 85.7% were single; 93.9% were relatively satisfied with their own major in the university. This research was approved by ethics committee of Southern Medical University.

### Procedures

Testing was conducted by well-trained psychology staff (one licensed doctoral-level psychology professor, one doctoral-level psychology student, and one master's-level psychology student). Participants self-completed the questionnaires during the 2018/2019 academic session anonymously. In order to access students in all disciplines, we chose classes that were compulsory general studies courses. The research team explained the purpose and the details of the process of the study at the end of the lectures. The students were duly informed that there would be no penalty for refusing to participate, and only eligible volunteers were included. The whole test, which lasted for about 30 min, needed to be finished online and participants could not submit their questionnaires unless they had finished. From our original sample of 2,640 eligible individuals, 25 students were excluded because they did not finish the questionnaires in 30 min.

### Measures

#### Meaningful Life Measure

The 23-item Meaningful Life Measure comprises five subscales, each designed to assess one of the following constituents of personal meaning: exciting life, purposeful life, principled life, accomplished life, valued life (Morgan and Farsides, 2009). The items are rated on a 7-point Likert scale, ranging from 1 (*totally disagree*) to 7 (*totally agree*), with higher scores representing stronger perceptions of meaning in life, and the potential total scores ranging from 23 to 161. The measure features seven reverse-scored items (items 4, 6, 9, 11, 17, 19, and 21).

“Exciting life” (items 1, 6, 11, 16, and 21) refers to an enthusiastic orientation that regards life as exciting, interesting, or engaging. Sample items include “Life to me seems… completely routine” (which would be rated 1 point) or “Life to me seems always exciting” (scoring a 7) and “My life interests and excites me.” “Purposeful life” (items 4, 9, 14, and 19) refers to an individual's sense of having clear goals and aims. Sample items include “I have a clear idea of what my future goals and aims are,” “In my life, I have no goals or aims at all” (scoring 1), and “In my life, I have very clear goals and aims” (scoring 7). “Principled life” (items 3, 8, 13, 18, and 23) measures the extent to which individuals have an appropriate personal philosophy or framework through which they understand and navigate life. Sample items include “I have a philosophy of life that really gives my living significance” and “I have a personal value system that makes my living worthwhile.” “Accomplished life” (items 2, 7, 12, 17, and 22) aims to assess an individual's sense that personal goals are being achieved or fulfilled. Sample items include “So far, I am pleased with what I have achieved in life” and “I have failed to accomplish much in life.” “Valued life” (items 5, 10, 15, and 20) evaluates a sense of intrinsic life value. Sample items include “My life is significant” and “I really value my life.”

The Chinese version of the Meaningful Life Measure (revised) demonstrated good reliability and validity. All of the items showed good discrimination indexes (*r* = 0.753–0.838, *p* < 0.001). The total internal consistency reliability was 0.942, and it ranged from 0.782 to 0.877 across the five dimensions; the total split-half reliability was 0.920, and it ranged from 0.752 to 0.830 in the five dimensions; and the total test–retest reliability was 0.871, and it ranged from 0.783 to 0.805 in the five dimensions (Xiao et al., 2017). Specific items can be found in Appendix A.

#### Suicidal Behaviors Questionnaire

Suicidal behavior was evaluated using the Chinese version of the Suicidal Behaviors Questionnaire (Linehan, 1981), consisting of four items on a Likert-type scale. Total index scores ranged from 3 to 18, with higher scores indicating higher suicide possibility. A score of 7 has been suggested as the cut-off point for the general population and a score of 8 for psychiatric inpatients to assess suicide risk levels clinically (Osman et al., 2001). Item 1 assesses lifetime suicidal ideation and attempts, item 2 captures the frequency of suicidal ideation in the past 12 months, item 3 evaluates the threat of suicide attempt, and item 4 corresponds to the self-reported likelihood of suicidal behavior. The Suicidal Behaviors Questionnaire (Chinese version) has been validated in general population samples, and strong evidence has been found for its psychometric properties. In a clinical sample its internal consistency was 0.88 and 0.87 in a non-clinical sample (Osman et al., 2001; Zhao et al., 2012).

#### Schizotypal Personality Questionnaire

The Schizotypal Personality Questionnaire (SPQ) screens for schizotypy or schizotypal personality disorder (Raine, 1991; Chen et al., 1997) through reference to 74 self-reported statements or questions. The scoring key of the SPQ is based on participants' positive or negative responses (“yes” = 1, “no” = 0). Scores range from 0 to 74, with each affirmatively endorsed item counting as 1 point and higher scores indicating higher levels of schizotypy. The SPQ also shows good reliability and validity with other measures of schizotypal personality traits (Raine, 1991). Its three-factor structure (cognitive-perceptual, interpersonal, disorganized) has acceptable cross-cultural validation and internal consistency, with a Cronbach's alpha of 0.82 for the cognitive-perceptual dimension, 0.84 for the interpersonal dimension, and 0.78 for the disorganized dimension (Raine et al., 1994). Sample items include “Do you sometimes feel that things you see on the TV or read in the newspaper have a special meaning for you?,” “Have you ever felt that you are communicating with another person telepathically (by mind-reading)?,” and “I often hear a voice speaking my thoughts aloud.”

### Data Analysis

Anonymized data were exported into SPSS (version 22) and AMOS (version 24.0) statistical software packages for analysis. *T*-test and χ^2^test were used to explore the significant demographic differences. We used *d* and *eta* to estimate the effect sizes in mean comparisons. Spearman's correlation was used to explore univariate associations between suicidal behavior, meaningfulness, and schizotypy. Simple mediation analysis was conducted to test the direct effect of two-path model and a three-path mediation model, while multi-group analysis was conducted to test the effects of gender differences on hypothesized model. The direct and indirect associations between schizotypy and suicidal behavior were examined using the structural equation model. Chi-square statistic (χ^2^), degrees of freedom (*df* ), normed fit index (NFI), comparative fit index (CFI), incremental fit index (IFI), and root mean square error of approximation (RMSEA) tests were performed to evaluate the fit of the structural equation model. The results (NFI > 0.9, CFI > 0.9, IFI > 0.9, and RMSEA < 0.05) indicated acceptable model fit.

## Results

### Correlations Among Study Variables

Skewness and kurtosis measurement results are shown in Table 1. The matrix of Spearman correlation coefficients presented in Table 2 shows that suicidality, meaningfulness, and schizotypy were found to be related to one another. In particular, meaningfulness, incorporating its five dimensions (exciting life, principled life, purposeful life, accomplished life, and valued life), was negatively correlated with suicidal behavior (*r* = −0.39, *p* < 0.01) and schizotypy (*r* = −0.32, *p* < 0.01). Moreover, suicidal behavior was positively associated with schizotypy (*r* = 0.26, *p* < 0.01) and its three subtypes (positive symptoms, negative symptoms, and disorganized symptoms). Additionally, age had no correlation with other variables. However, as shown in Table 3, females were found to be more likely to have suicidal attempts or ideas than males (*p* < 0.001) and males scored significantly higher than females in disorganized schizotypy, which indicates that gender could have an influence on the potential mediating effect. Around 10.6% of participants showed a high level of suicide risk.

**Table 1:** Descriptive characteristics of measured variables.

	**$\bar{X}\pm$ SD**	**Skewness**	**Kurtosis**
Suicidality	4.46 ± 1.57	1.96	5.31
Meaningfulness	133.29 ± 17.79	−0.94	1.12
Schizotypy	14.96 ± 9.42	0.99	0.93
Purposeful life	5.58 ± 1.20	−0.95	0.36
Valued life	6.41 ± 0.69	−1.96	5.72
Accomplished life	5.56 ± 0.95	−0.89	0.96
Principled life	5.78 ± 0.94	−1.04	1.32
Exciting life	5.73 ± 0.91	−0.88	0.82
Cognitive-perceptual	6.93 ± 4.17	0.95	1.21
Interpersonal	5.56 ± 5.02	1.26	1.56
Disorganized	3.25 ± 2.94	1.38	1.75
Item 1	1.54 ± 0.66	1.18	1.74
Item 2	1.20 ± 0.54	3.15	11.27
Item 3	1.18 ± 0.44	2.42	5.31
Item 4	0.53 ± 0.79	1.92	4.75
Age	18.35 ± 0.81	1.13	4.17
Gender	1,020 (male)	0.451	−1.798

*Items 1–4 are the four items of SBQ. Item 1: “Have you ever thought about or attempted to kill yourself?”; Item 2: “How often have you thought about killing yourself in the past year?”; Item 3: “Have you ever told someone that you were going to commit suicide, or that you might do it?”; Item 4: “How likely is it that you will attempt suicide someday?”*.

**Table 2:** Correlations among measured variables.

	**Suicidality(d)**	**Meaningfulness(d)**	**Schizotypy(d)**
Suicidality	1		
Meaningfulness	−0.39^**^	1	
Schizotypy	0.26^**^	−0.32^**^	1
Purposeful life	−0.29^**^	0.80^**^	−0.28^**^
Valued life	−0.40^**^	0.75^**^	−0.21^**^
Accomplished life	−0.32^**^	0.83^**^	−0.27^**^
Principled life	−0.28^**^	0.85^**^	−0.23^**^
Exciting life	−0.34^**^	0.84^**^	−0.31^**^
Cognitive-perceptual	0.18^**^	−0.13^**^	0.81^**^
Interpersonal	0.25^**^	−0.38^**^	0.86^**^
Disorganized	0.23^**^	−0.29^**^	0.81^**^
Item 1	0.80^**^	−0.28^**^	0.21^**^
Item 2	0.57^**^	−0.28^**^	0.14^**^
Item 3	0.54^**^	−0.15^**^	0.09^**^
Item 4	0.69^**^	−0.34^**^	0.18^**^
Age	−0.02	−0.011	0.001
Gender	5.18^**^ (0.21)	−0.56 (−0.02)	−0.87 (−0.03)

** *p < 0.01, items 1–4 are the four items of SBQ. Item 1: “Have you ever thought about or attempted to kill yourself?”; Item 2: “How often have you thought about killing yourself in the past year?”; Item 3: “Have you ever told someone that you were going to commit suicide, or that you might do it?”; Item 4: “How likely is it that you will attempt suicide someday?”*.

**Table 3:** Different performance in males and female.

	**Male**	**Female**	***T*-test/*χ^*2*^ (d/eta)***	***p***	**Percentage in total sample**
Schizotypy	15.15	14.82	−0.87 (− 0.03)	0.723	
Cognitive-perceptual	6.82	6.99	1.02 (0.042)	0.31	
Interpersonal	5.66	5.50	−0.82 (−0.03)	0.41	
Disorganized	3.51	3.07	−3.75 (−0.03)	<0.001^**^	
Meaningfulness	133.56	133.16	−0.56 (−0.02)	0.159	
Suicidality	4.23	4.59	5.18 (0.21)	<0.001^**^	
SBQ-R≥7	81	197	12.735 (0.07)	<0.001^**^	10.6%

** *p < 0.01; “Cognitive-perceptual,” “Interpersonal,” and “Disorganized” were subscales of Schizotypal Personality Questionnaire; SBQ-R represents Suicidal Behaviors Questionnaires-Revised; SBQ-R≥7 means non-clinic high suicide risk*.

### Structural Model

#### Two-Factor Model From Schizotypy to Suicidality in All Samples

**Figure 1:**
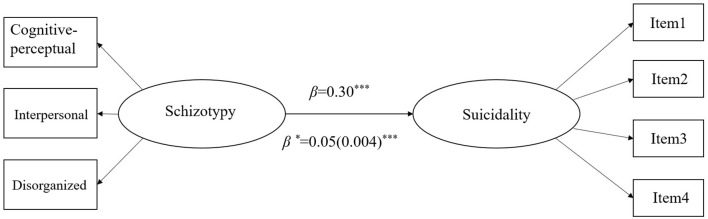
Path Model from schizotypy to suicidality in all samples. ***P < 0.001, β, standardized coefficients; β*, unstandardized coefficients; SE, standard errors in brackets.

The overall fit of the two-factor model was found to be acceptable (χ^2^ = 90.688, *df* = 13, IFI = 0.982, CFI = 0.982, NFI = 0.979, RMSEA = 0.047, 90%IC RMSEA = 0.039–0.057). Figure 1 shows the result of the path analysis and that the direct effect of schizotypy on suicidal behavior was significant (standardized pathway coefficient = 0.30, unstandardized pathway coefficient = 0.05, *SE* = 0.004, *p* < 0.001).

#### Three-Factor Model Between Schizotypy and Suicidal Behavior Through Meaningfulness in All Samples

**Figure 2:**
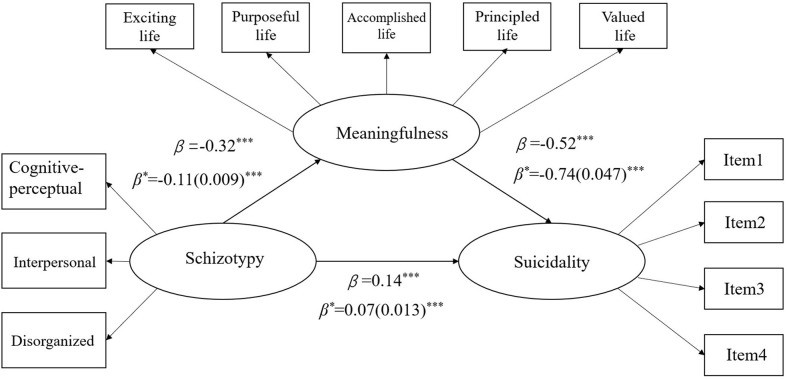
Mediation model for relationship among schizotypy, meaningfulness, and suicidality in all samples. ***p < 0.001, β, standardized coefficients; β*, unstandardized coefficients; SE, standard errors in brackets.

All the pathways were found to be significant, and the appropriately modified three-factor model was found to have a good model fit (χ^2^ = 222.53, *df* = 43, IFI = 0.99, CFI = 0.99, NFI = 0.98, RMSEA = 0.04, 90%IC RMSEA = 0.035–0.045). As shown in Figure 2, the indirect effect from schizotypy to suicidality through meaningfulness, which was calculated through multiplication of the two indirect standardized pathway coefficients, was statistically significant, and so was the direct pathway (standardized pathway coefficient = 0.14, *p* < 0.001). This result established that a partial mediating effect of meaningfulness existed, and this was presented in Figure 2. The unstandardized coefficients and its SE of pathways are also depicted in Figure 2. Also, the final model held true for all three schizotypy subscales and the results can be found in Appendix B.

Furthermore, around 55.47% of the schizotypy and suicidality relationship can be explained by the mediating effect of meaningfulness.

#### Three-Factor Model Between Schizotypy and Suicidal Behavior Through Meaningfulness in Males vs. Females: Multi-Group Analysis

**Figure 3:**
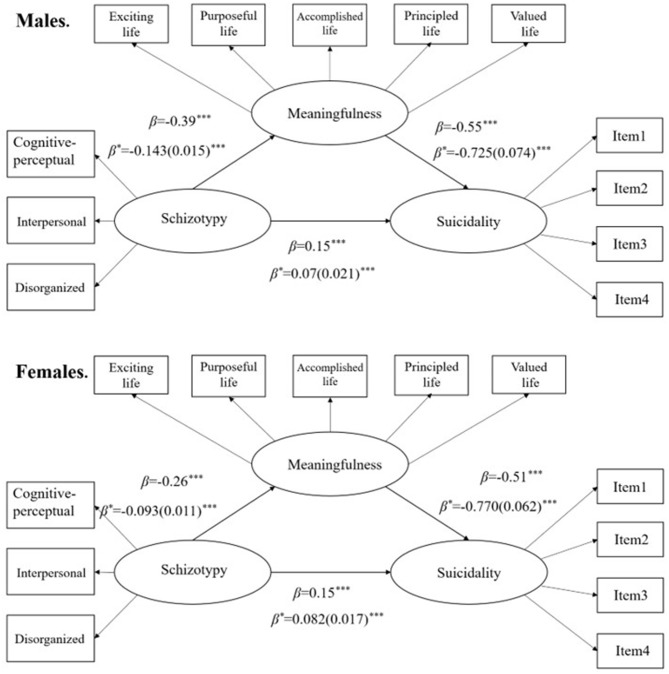
Mediation model for relationship among schizotypy, meaningfulness, and suicidality in males (upper half) and females (lower half). ***p < 0.001, β, standardized coefficients; β*, unstandardized coefficients; SE, standard errors in brackets.

In order to control the influence on irrelevant variables (i.e., gender), multi-group analyses were conducted to assess whether gender might change the mediating effect of meaningfulness between suicidality and schizotypy. We found that the model fit of unconstrained model in both gender group was adequate (see Table 4), χ^*2*^ = 282.95, *df* = 86, IFI = 0.99, CFI = 0.98, NFI = 0.98, RMSEA = 0.03, 90%IC RMSEA = 0.026–0.033. The measurement weights model provided good model fit (χ^*2*^ = 306.99, *df* = 96, IFI = 0.98, CFI = 0.98, NFI = 0.98, RMSEA = 0.03, 90%IC RMSEA = 0.026–0.033), which was significantly changed compared with that of the unconstrained model (Δχ^*2*^ = 24.04, Δ*df* =10, *p* < 0.01). The structural weights model also yielded good model fit, χ^*2*^ = 320.44, *df* = 99, IFI = 0.98, CFI = 0.98, NFI = 0.97, RMSEA = 0.03, 90%IC RMSEA = 0.026–0.033, which was significantly changed as well (Δχ^*2*^ = 37.50, Δ*df* = 13, *p* < 0.001). Although all the three models had different data-model fit, the significance of all the pathways in both male and female groups were consistent with the three-factor model in all samples (see Figure 3). These results revealed that gender did not have significant effects on the mediation of meaningfulness in schizotypy-suicidality relationship.

**Table 4:** Goodness of fit indexes for model comparisons.

	***χ^*2*^***	***Df***	***χ^*2*^*/*df***	***Δχ^*2*^***	***Δdf***	***p***	**IFI**	**CFI**	**NFI**	**RMSEA**
Threshold			<5				≥0.90	≥0.90	≥0.90	<0.05
Unconstrained model	282.95	86	3.29				0.98	0.98	0.98	0.03
Measurement weights model	306.99	96	3.20	24.04	10	<0.01^**^	0.98	0.98	0.98	0.03
Structural weights model	320.44	99	3.24	37.50	13	<0.001^**^	0.98	0.98	0.97	0.03

** *p < 0.01, χ^2^, chi-square; df, degree of freedom; IFI, Incremental fit index; CFI, Comparative fit index; NFI, Normed fit index; RMSEA, Root mean square error of approximation*.

## Discussion

The present study found a relationship among suicidality, meaningfulness, and schizotypy. Meaningfulness was found to be inversely associated with the other two factors and schizotypy was positively related with suicidality. What is more, the correlation between schizotypy and suicidality was substantially attenuated when meaningfulness was included, which may suggest that meaningfulness can partly mediate this relationship. Male and female participants showed different results regarding suicidality but gender did not have significant effects on the mediator effect of meaningfulness in schizotypy-suicidality relationship.

In line with previous studies, there was found to be a negative association between meaningfulness and suicidal behavior (Orbach et al., 2003; Bjerkeset et al., 2010; Wilchek-Aviad, 2015) and a positive correlation between schizotypy and suicidal behavior (Joiner et al., 2001; Kwapil et al., 2013; Teraishi et al., 2014; Jahn et al., 2016). Based on the assumption that individuals with high suicide risk question their life's value and have lost the desire to find their life's meaning, it is thought that people with less meaningfulness are at increased risk of having serious suicidal thoughts and attempts (Zhao et al., 2012). Additionally, people with schizotypy can have symptoms such as anhedonia (Meehl, 1962; Kwapil et al., 2013), which can make people unable to feel happiness and to hurt themselves or even commit suicide. There are gender differences in respect to suicidality, which has been proved by many studies. Although suicide brings more death to males, females register many more unsuccessful attempts (Sava and Papari, 2015), which is consistent with our finding. Compared with males, female suicide attempters are more likely to be influenced by social and interpersonal factors (Sun and Zhang, 2017), which is a key issue for people with schizotypal traits.

Nonetheless, the negative relationship between schizotypy and meaningfulness has to date received relatively little attention. Meaningfulness depends on culture and social connections, especially positive interactions with others (Baumeister et al., 2013; Lambert et al., 2013), while the social deficits of schizotypy (Triebwasser et al., 2012) and the “overall crisis of common sense” of these individuals (Stanghellini and Ballerini, 2006) greatly reduce their sense of meaning in life. The three-factor structure in the SPQ (Raine et al., 1994) indicates that people with schizotypal traits can have some psychotic-like symptoms, such as unusual perceptual experiences, paranoid ideation, and thoughts and behavior disturbances (Kwapil et al., 2018). Schizotypal adults maintain beliefs of conspiracy, irrational fear, or suspiciousness, which result in social anxiety, a lack of close friends, and other interpersonal problems. Moreover, these cognitive-perceptual, interpersonal, and disorganized features of schizotypy may result in a child having negative experiences early in the developmental process, such as verbal abuse and social exclusion (Raine et al., 2011; Wong and Raine, 2018). Consequently, peer victimization (Raine et al., 2011; Lam et al., 2016), and childhood trauma (Kelleher et al., 2008; Velikonja et al., 2015) caused by schizotypal features could give rise to a lack of meaningfulness. Additionally, individuals with an inability to experience pleasure, including a reduction in experiencing emotions overall or an increased sensitivity to negative emotions (Smith et al., 1995), could also experience a negative impact on their sense of enthusiasm, goals, and self-value. Therefore, with all the types of schizotypal personality, individuals' cognitive and behavioral dysfunctions can destroy their capability to understand life and achieve success. These individuals typically find less meaning and hope in life, have a lower quality of life, and may be at increased risk of experiencing mental health problems (Alminhana et al., 2017).

The present study found a direct effect of schizotypy on suicide behaviors, which is consistent with results from previous studies (Joiner et al., 2001; Kwapil et al., 2013), indicating that schizotypy may be a clinically useful indicator of suicide risk (Jahn et al., 2016). However, given the biological homology of schizotypy and schizophrenia (Triebwasser et al., 2012), it is not easy to alter these traits, which makes it difficult to effectively intervene in the suicide risk of schizotypal individuals. Our study found that meaningfulness mediates between schizotypy and suicidal behaviors, suggesting that, when it is difficult to directly change schizotypal personality traits, it may be possible to manage suicide risk by improving these individuals' sense of meaning in life. Interpersonal alienation, which affects such a sense of meaning, can be addressed through social skills training and group therapy (Mckay and Neziroglu, 1996). Methods that do not involve the pathological defects of schizotypy, such as promoting self-expression, integration, and reflection in the temporal dimension (Baumeister et al., 2013), also contribute to a sense of meaning. The specific cognitive style of schizotypy also suggests the potential value of initiatives such as creative activities and religious/spiritual well-being (Burch et al., 2006; Unterrainer et al., 2011). Further research is needed to determine whether these methods of enhancing sense of meaning are useful for schizotypal individuals.

Several methodological limitations of the present study should be acknowledged. First, as our research was of a cross-sectional design, a causal relationship cannot be established between meaningfulness and suicidality. Second, measurements used in our study were self-reported. Therefore, recall biases and reporting biases of some questions regarding suicidality might exist. Third, other confounding variables and background variables need to be considered or added into this model, such as a history of childhood abuse, depressive symptoms, and symptoms of anxiety. What is more, meaningfulness, as a potential mediator, requires an experimental study design and additional alternative approaches in order to obtain further confirmation. Despite these limitations, though, the current study proposes a possible mediator in the schizotypy–suicidality relationship, which suggests that assessing meaningfulness could provide more detailed information about suicide risk in individuals with schizotypy. Additionally, it may be an effective intervention as reducing suicide risk among people with schizotypy to improve their meaningfulness. Thus, our findings provide important insights into the development of life education and psychological crisis intervention in colleges and universities. However, there are several other risk factors that have not been tested yet, and no prior studies have comprehensively tested all well-established risk factors in a single model, in respect of which further studies are expected in the future.

## Data Availability Statement

The datasets generated for this study are available on request to the corresponding author.

## Ethics Statement

The studies involving human participants were reviewed and approved by Southern Medical University Ethics Committee. The patients/participants provided their written informed consent to participate in this study.

## Author Contributions

JZ and XZ designed the experiment and revised the manuscript. SL and DL have contributed equally to the data organization, statistical analysis, and the first draft of the manuscript. All authors approved the final version of the manuscript.

### Conflict of Interest

The authors declare that the research was conducted in the absence of any commercial or financial relationships that could be construed as a potential conflict of interest.
