# Supplementary material for: The Mediator Effect of Meaningfulness on the Relationship Between Schizotypy Traits and Suicidality
Source: Front Psychol. 2020 Mar 24;11:493. doi: 10.3389/fpsyg.2020.00493 (PMC7179699; doi:10.3389/fpsyg.2020.00493)
Supplement: Supplementary file 1 [file Data_Sheet_1.docx]

**Appendix A**

The Meaningful Life Measure-Chinese Revised （MLM-CR）

指导语：请仔细阅读以下是陈述，并根据实际情况圈出最符合您实际情况的数字。除非另有说明，请按以下表格作答。

Instruction: Please read each of the following statements carefully and then circle the appropriate

number to indicate your opinion. Please answer according to the scale below, unless otherwise stated.

|  |  | 非常不同意  Strongly disagree | 比较不同意  Disagree | 有点不同意  Slightly disagree | 中性  Neither agree or disagree | 有点同意  Slightly agree | 比较同意  Agree | 非常同意  Strongly agree |
| --- | --- | --- | --- | --- | --- | --- | --- | --- |
| 1 | **生活对我来说总是令人兴奋的。**  **Life to me seems always exciting.** | 1 | 2 | 3 | 4 | 5 | 6 | 7 |
| 2 | **回想人生中已取得的成绩，令我感到满意。**  **I find it satisfying to think about what I have accomplished in life.** | 1 | 2 | 3 | 4 | 5 | 6 | 7 |
| 3 | **我有自己一套生活方式与体系使我能更好地感受生活。**  **I have a system or framework that allows me to truly understand my being alive.** | 1 | 2 | 3 | 4 | 5 | 6 | 7 |
| 4 | **在我的生活中毫无目标或计划。**  **In my life I have no goals or aims at all.** | 1 | 2 | 3 | 4 | 5 | 6 | 7 |
| 5 | **我的人生是有价值的。**  **My life is worthwhile.** | 1 | 2 | 3 | 4 | 5 | 6 | 7 |
| 6 | **我觉得每天都是一成不变的。**  **Every day is exactly the same.** | 1 | 2 | 3 | 4 | 5 | 6 | 7 |
| 7 | **到目前为止，我对生活中已达成的目标感到满意。**  **So far, I am pleased with what I have achieved in life.** | 1 | 2 | 3 | 4 | 5 | 6 | 7 |
| 8 | **我的人生观真正赋予了我生活的意义。**  **I have a philosophy of life that really gives my living significance.** | 1 | 2 | 3 | 4 | 5 | 6 | 7 |
| 9 | **我还没有发现人生的目的和使命。**  **I have discovered no mission or purpose in life.** | 1 | 2 | 3 | 4 | 5 | 6 | 7 |
| 10 | **我的人生是有意义的。**  **My life is significant.** | 1 | 2 | 3 | 4 | 5 | 6 | 7 |
| 11 | **面对日常的任务和工作,令我感到痛苦和厌烦。**  **Facing my daily tasks is a painful and boring experience.** | 1 | 2 | 3 | 4 | 5 | 6 | 7 |
| 12 | **在某些事情上我非常成功。**  **I have been very successful in achieving certain things.** | 1 | 2 | 3 | 4 | 5 | 6 | 7 |
| 13 | **我的个人价值观使我的生活具有价值感。**  **I have a personal value system that makes my living worthwhile.** | 1 | 2 | 3 | 4 | 5 | 6 | 7 |
| 14 | **我对自己未来的目标和计划有明确的认识。**  **I have a clear idea of what my future goals and aims are.** | 1 | 2 | 3 | 4 | 5 | 6 | 7 |
| 15 | **我很珍惜我的生命。**  **I really value my life.** | 1 | 2 | 3 | 4 | 5 | 6 | 7 |
| 16 | **我的生活富有乐趣。**  **My life interests and excites me.** | 1 | 2 | 3 | 4 | 5 | 6 | 7 |
| 17 | **我在生活的很多方面都很失败。**  **I have failed to accomplish much in life.** | 1 | 2 | 3 | 4 | 5 | 6 | 7 |
| 18 | **我的世界观使我能理解自我存在的意义。**  **The beliefs I hold about the world enable me to make sense out of my existence.** | 1 | 2 | 3 | 4 | 5 | 6 | 7 |
| 19 | **我的生活缺乏目标或方向。**  **I tend to wander aimlessly through life, without much sense of purpose or direction.** | 1 | 2 | 3 | 4 | 5 | 6 | 7 |
| 20 | **我高度尊重自我的生命。**  **I hold my own life in high regard.** | 1 | 2 | 3 | 4 | 5 | 6 | 7 |
| 21 | **我的日常生活单调乏味。**  **My daily living is dull and routine.** | 1 | 2 | 3 | 4 | 5 | 6 | 7 |
| 22 | **想起生活中那些已经完成的事情，令我感觉良好。**  **I feel good when I think of the things I have accomplished in life.** | 1 | 2 | 3 | 4 | 5 | 6 | 7 |
| 23 | **我所持有的价值观使我的生活更富有意义。**  **I hold certain values which I feel greatly enrich my life with significance.** | 1 | 2 | 3 | 4 | 5 | 6 | 7 |

**Appendix B**

Three-factor model between SPQ subscales and suicidal behavior through meaningfulness in all samples


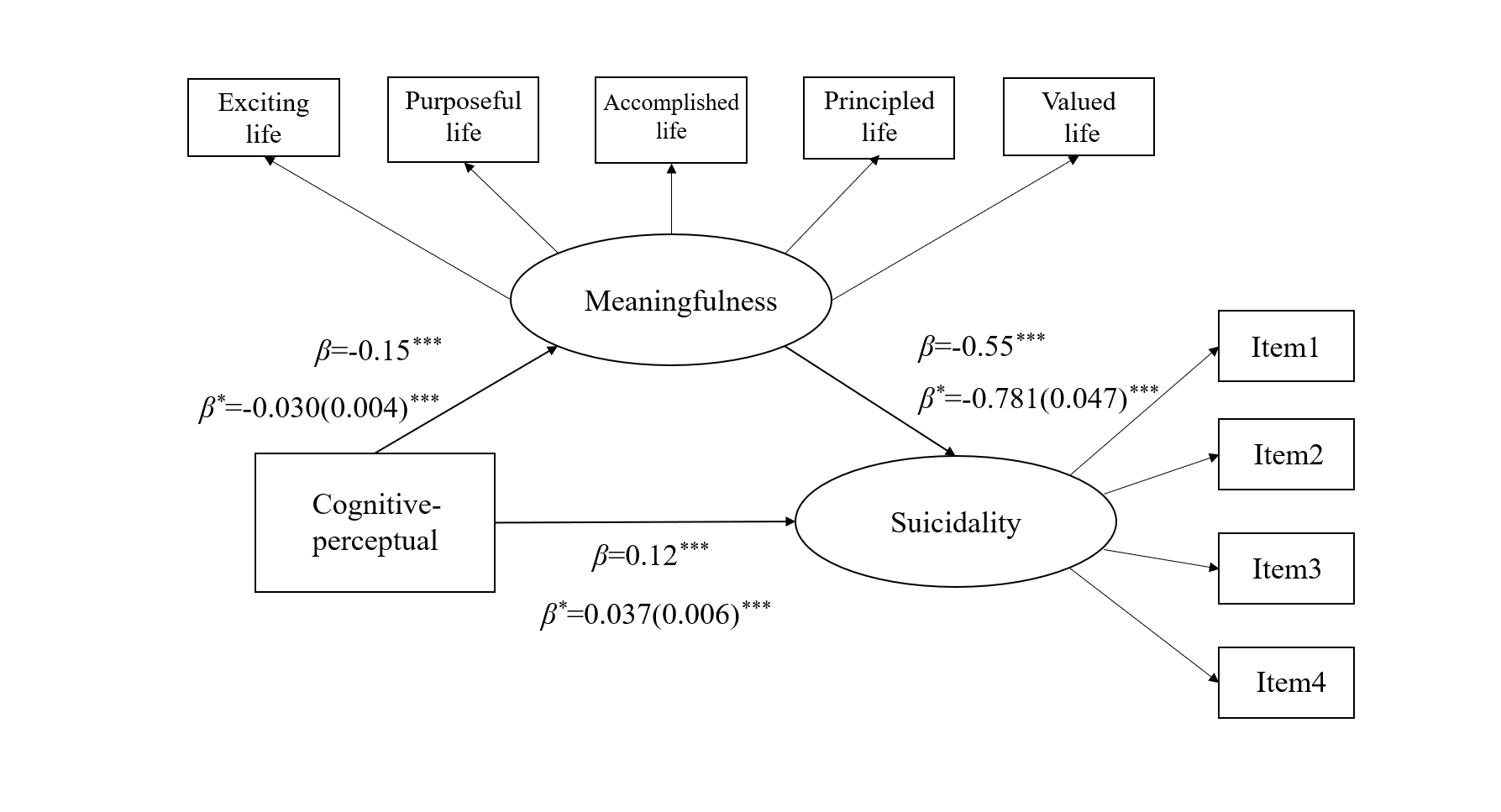


**Figure S1. Three-factor model between cognitive-perceptual schizotypy and suicidal behavior through meaningfulness in all samples**

Note. ****p*＜0.001, *β*=standardized coefficients, *β*=*unstandardized coefficients, SE=standard errors in brackets


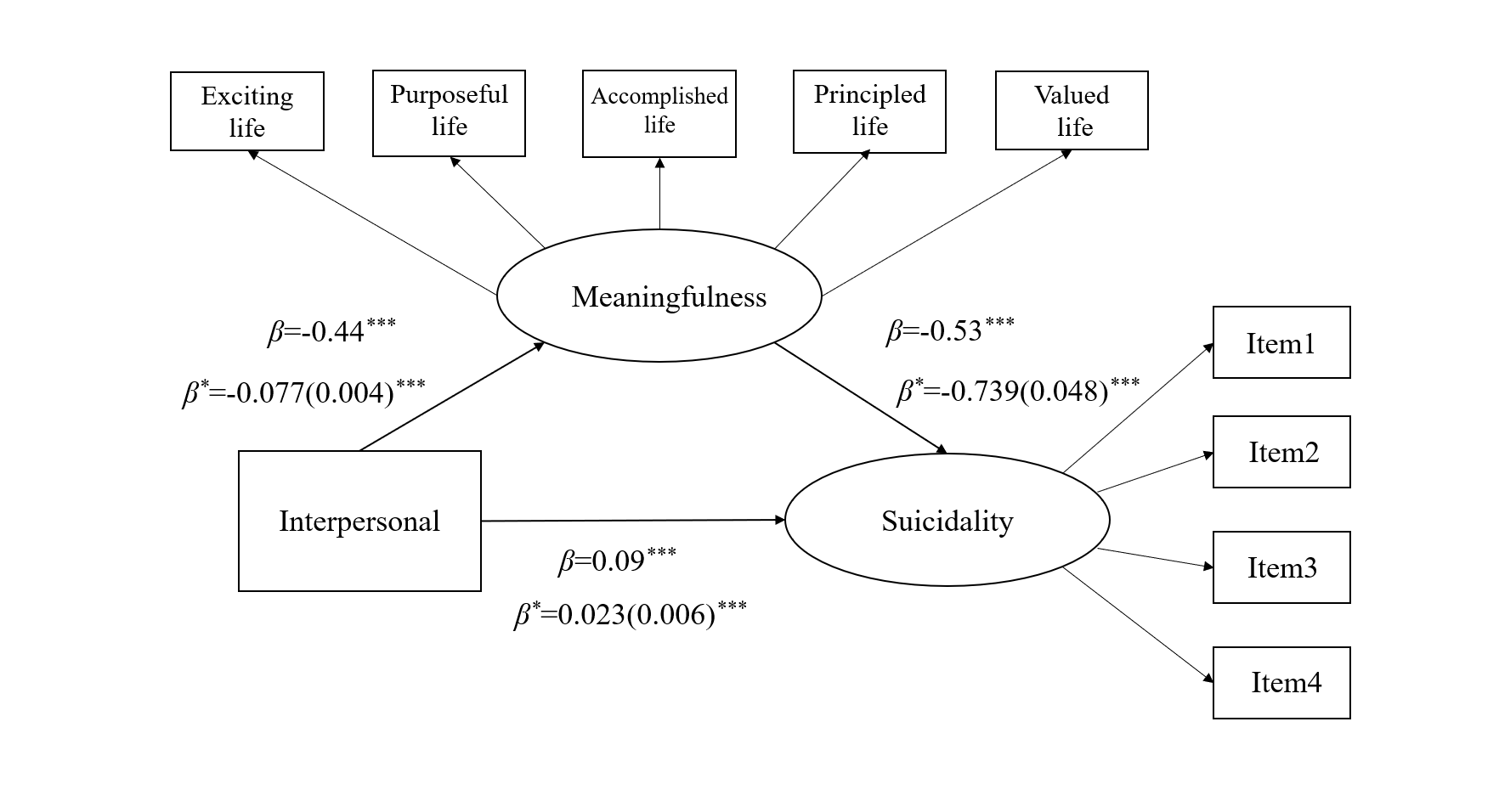


**Figure S2. Three-factor model between interpersonal schizotypy and suicidal behavior through meaningfulness in all samples**

Note. ****p*＜0.001, *β*=standardized coefficients, *β*=*unstandardized coefficients, SE=standard errors in brackets


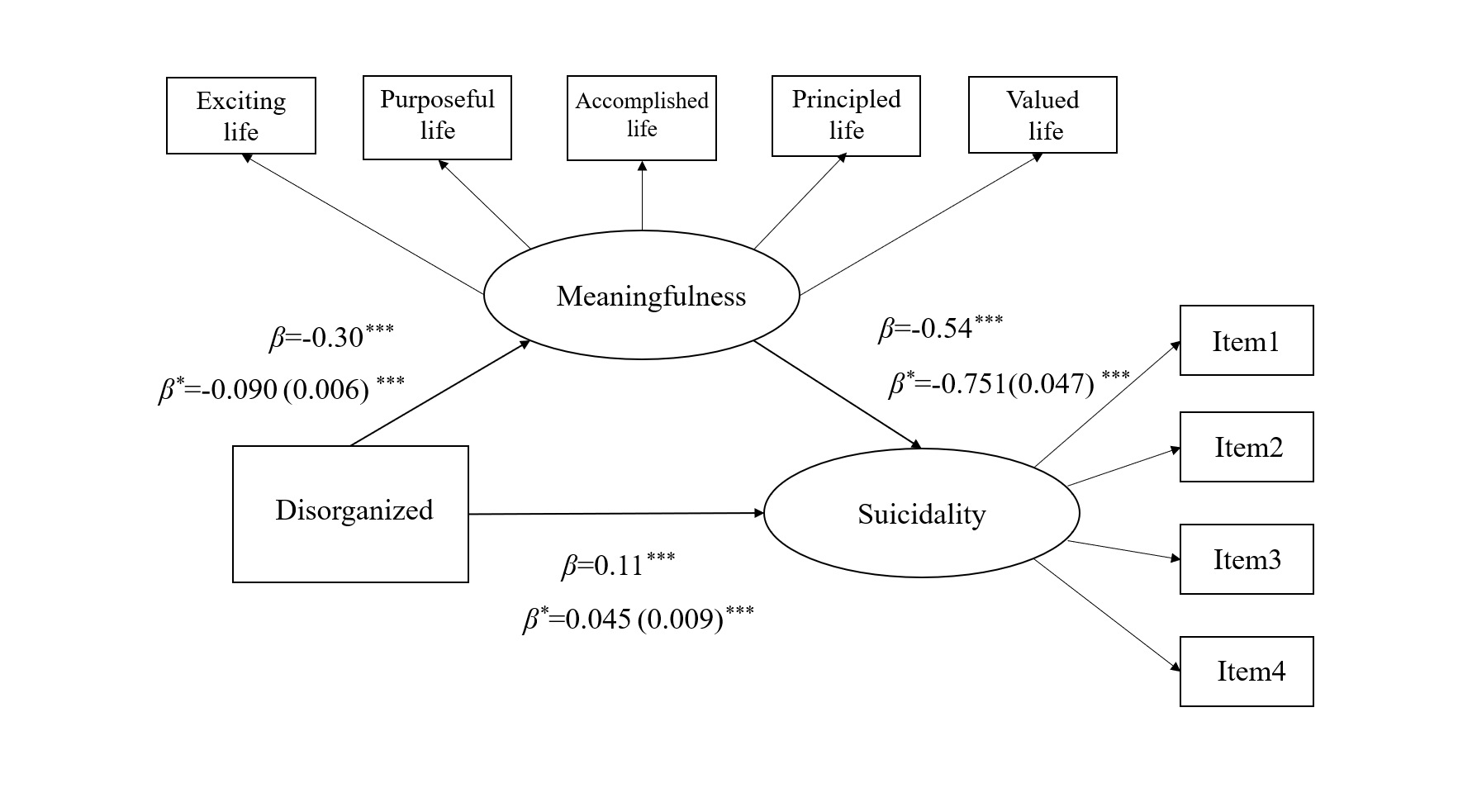


**Figure S3. Three-factor model between disorganized schizotypy and suicidal behavior through meaningfulness in all samples**

Note. ****p*＜0.001, *β*=standardized coefficients, *β*=*unstandardized coefficients, SE=standard errors in brackets
